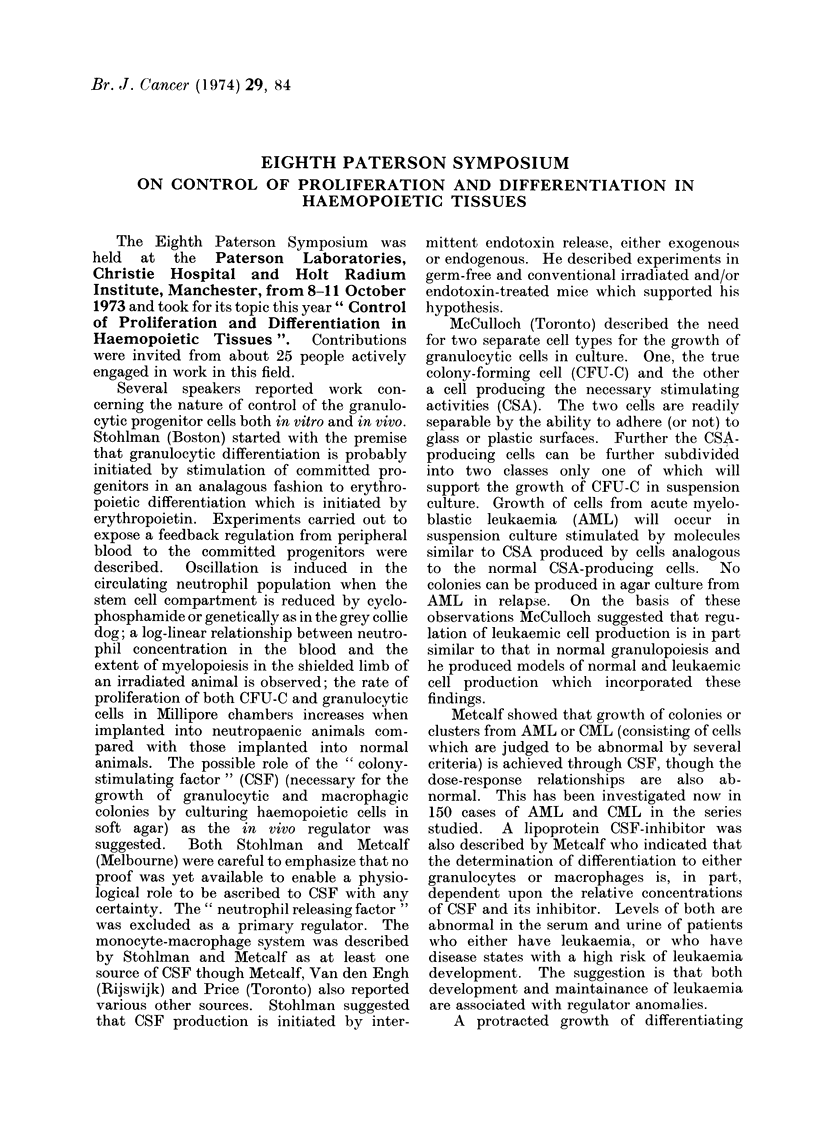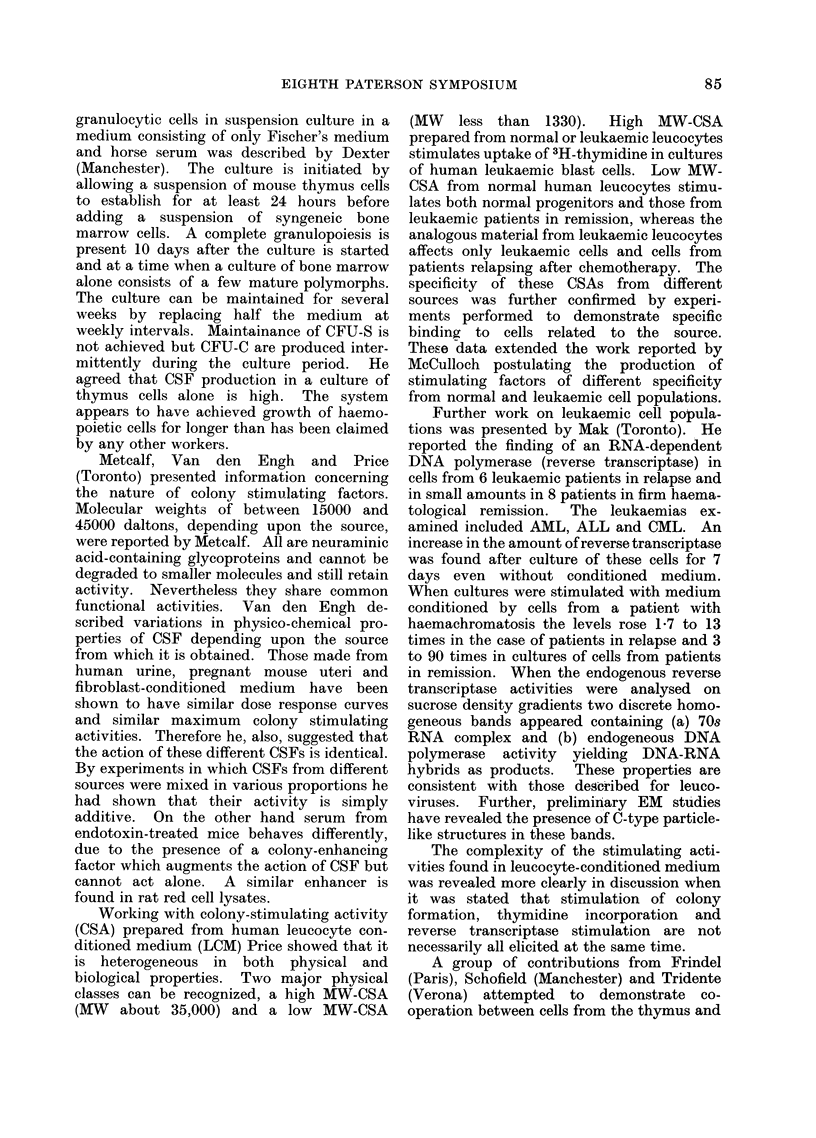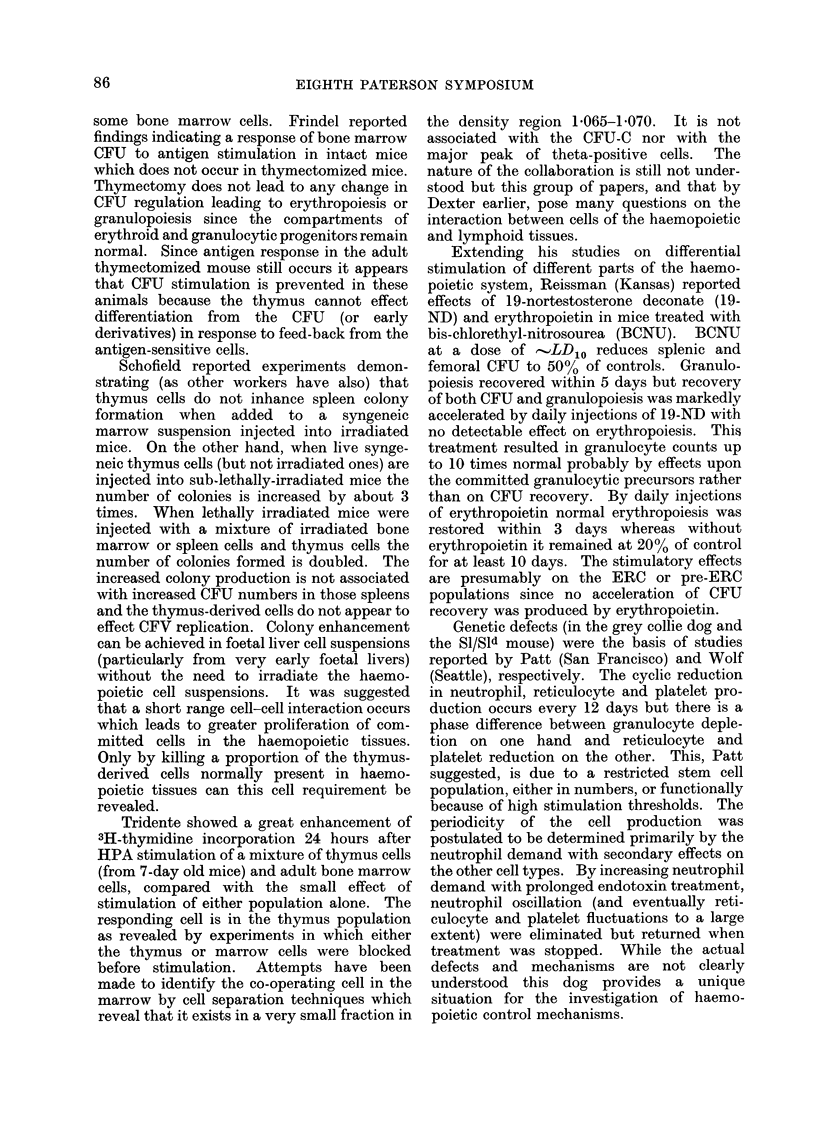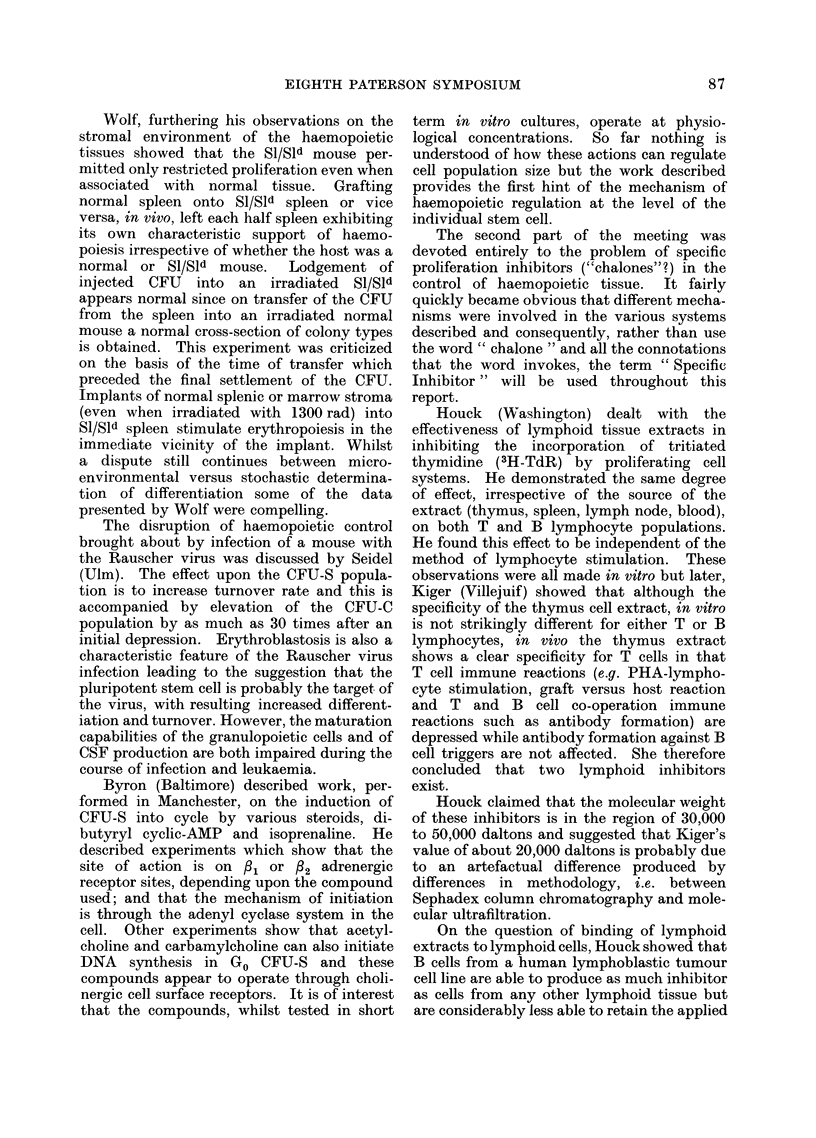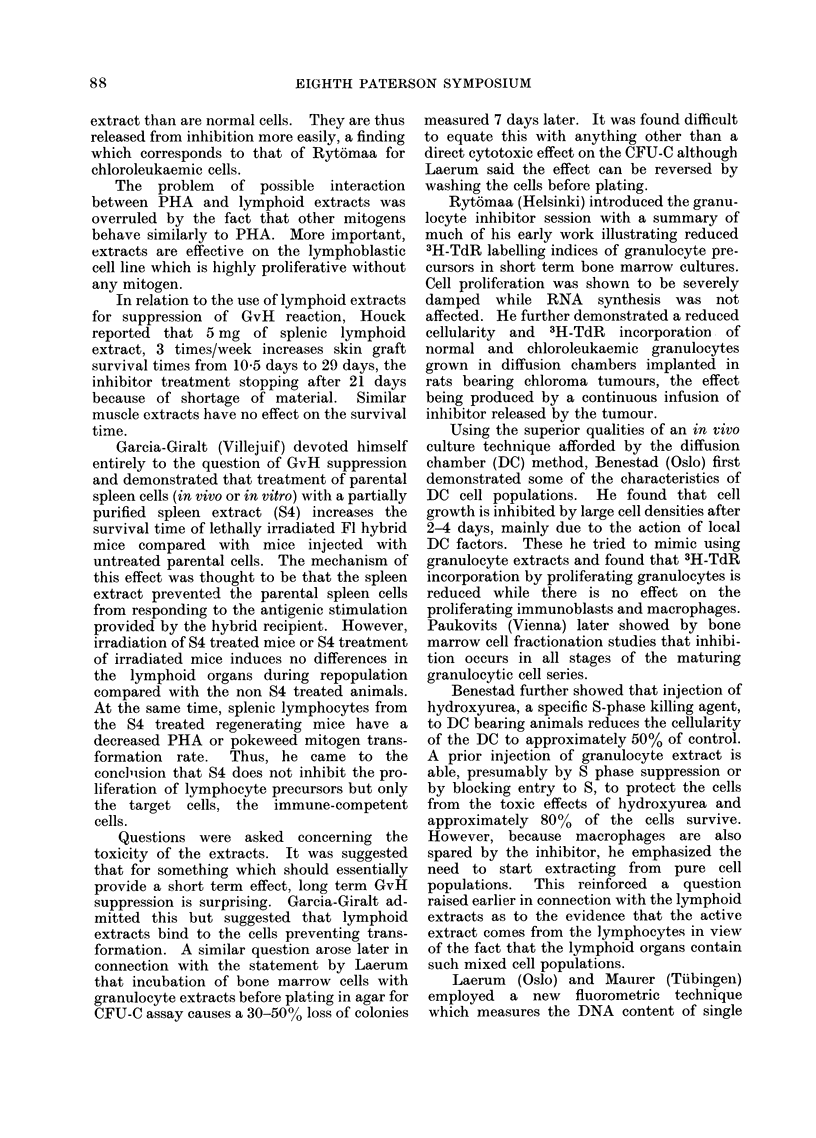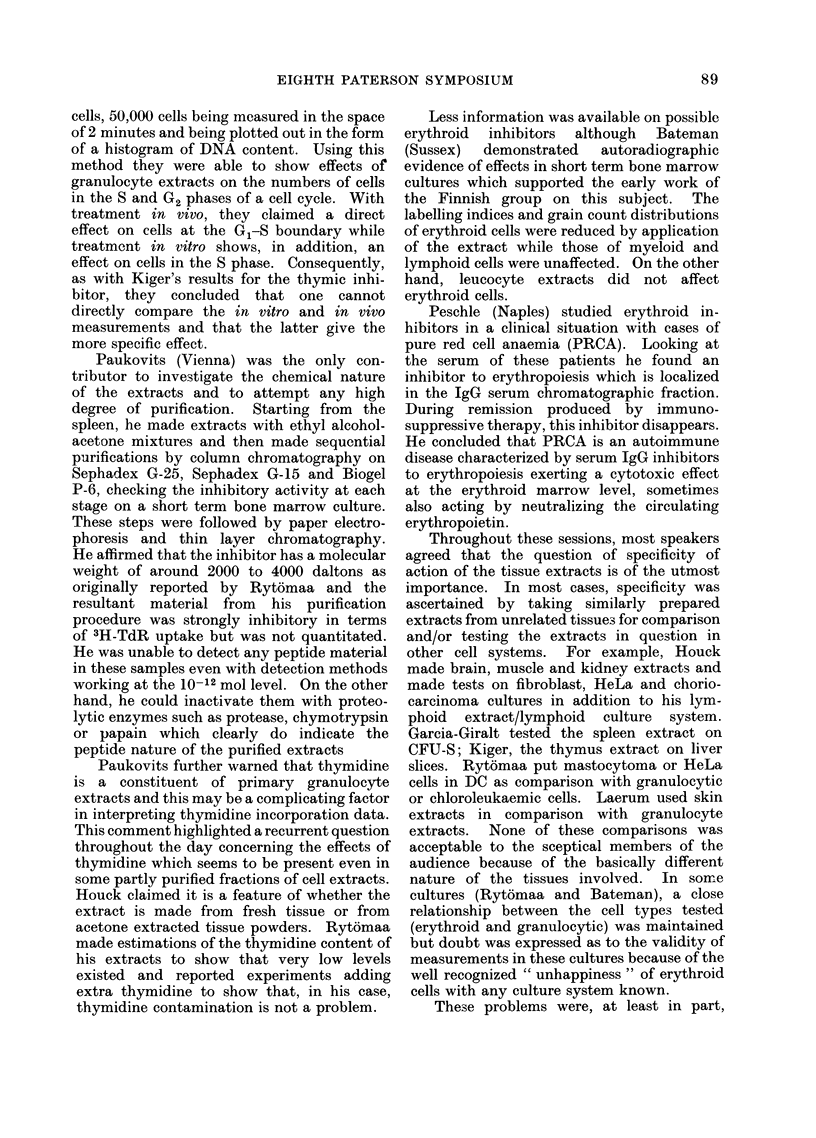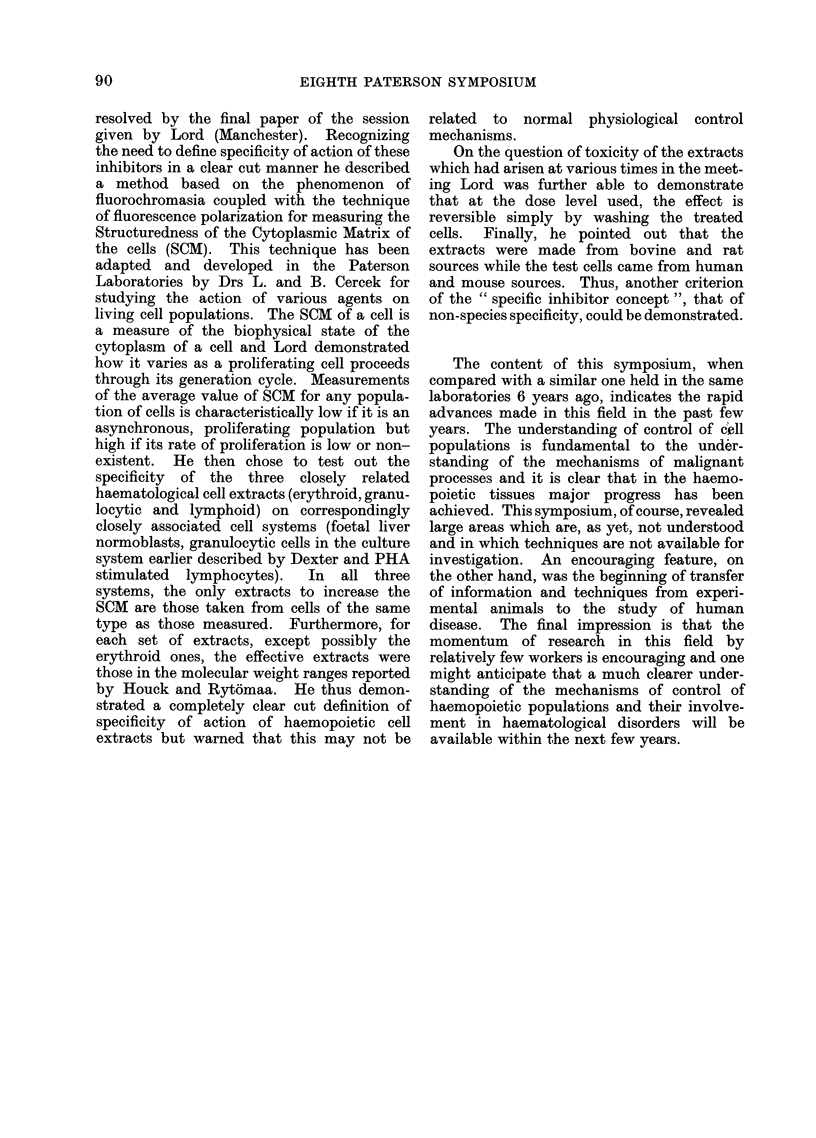# Eighth Paterson Symposium on Control of Proliferation and Differentiation in Haemopoietic Tissues

**Published:** 1974-01

**Authors:** 


*Br. J. Cancer* (1974) **29**, 84

### EIGHTH PATERSON SYMPOSIUM
### ON CONTROL OF PROLIFERATION AND DIFFERENTIATION IN HAEMOPOIETIC TISSUES

The Eighth Paterson Symposium was held at the **Paterson Laboratories, Christie Hospital and Holt Radium Institute, Manchester, from 8–11 October 1973** and took for its topic this year " **Control of Proliferation and Differentiation in Haemopoietic Tissues** ". Contributions were invited from about 25 people actively engaged in work in this field.

Several speakers reported work concerning the nature of control of the granulocytic progenitor cells both *in vitro* and *in vivo*. Stohlman (Boston) started with the premise that granulocytic differentiation is probably initiated by stimulation of committed progenitors in an analagous fashion to erythropoietic differentiation which is initiated by erythropoietin. Experiments carried out to expose a feedback regulation from peripheral blood to the committed progenitors were described. Oscillation is induced in the circulating neutrophil population when the stem cell compartment is reduced by cyclophosphamide or genetically as in the grey collie dog; a log-linear relationship between neutrophil concentration in the blood and the extent of myelopoiesis in the shielded limb of an irradiated animal is observed; the rate of proliferation of both CFU-C and granulocytic cells in Millipore chambers increases when implanted into neutropaenic animals compared with those implanted into normal animals. The possible role of the " colony-stimulating factor " (CSF) (necessary for the growth of granulocytic and macrophagic colonies by culturing haemopoietic cells in soft agar) as the *in vivo* regulator was suggested. Both Stohlman and Metcalf (Melbourne) were careful to emphasize that no proof was yet available to enable a physiological role to be ascribed to CSF with any certainty. The " neutrophil releasing factor " was excluded as a primary regulator. The monocyte-macrophage system was described by Stohlman and Metcalf as at least one source of CSF though Metcalf, Van den Engh (Rijswijk) and Price (Toronto) also reported various other sources. Stohlman suggested that CSF production is initiated by intermittent endotoxin release, either exogenous or endogenous. He described experiments in germ-free and conventional irradiated and/or endotoxin-treated mice which supported his hypothesis.

McCulloch (Toronto) described the need for two separate cell types for the growth of granulocytic cells in culture. One, the true colony-forming cell (CFU-C) and the other a cell producing the necessary stimulating activities (CSA). The two cells are readily separable by the ability to adhere (or not) to glass or plastic surfaces. Further the CSA-producing cells can be further subdivided into two classes only one of which will support the growth of CFU-C in suspension culture. Growth of cells from acute myeloblastic leukaemia (AML) will occur in suspension culture stimulated by molecules similar to CSA produced by cells analogous to the normal CSA-producing cells. No colonies can be produced in agar culture from AML in relapse. On the basis of these observations McCulloch suggested that regulation of leukaemic cell production is in part similar to that in normal granulopoiesis and he produced models of normal and leukaemic cell production which incorporated these findings.

Metcalf showed that growth of colonies or clusters from AML or CML (consisting of cells which are judged to be abnormal by several criteria) is achieved through CSF, though the dose-response relationships are also abnormal. This has been investigated now in 150 cases of AML and CML in the series studied. A lipoprotein CSF-inhibitor was also described by Metcalf who indicated that the determination of differentiation to either granulocytes or macrophages is, in part, dependent upon the relative concentrations of CSF and its inhibitor. Levels of both are abnormal in the serum and urine of patients who either have leukaemia, or who have disease states with a high risk of leukaemia development. The suggestion is that both development and maintainance of leukaemia are associated with regulator anomalies.

A protracted growth of differentiating granulocytic cells in suspension culture in a medium consisting of only Fischer's medium and horse serum was described by Dexter (Manchester). The culture is initiated by allowing a suspension of mouse thymus cells to establish for at least 24 hours before adding a suspension of syngeneic bone marrow cells. A complete granulopoiesis is present 10 days after the culture is started and at a time when a culture of bone marrow alone consists of a few mature polymorphs. The culture can be maintained for several weeks by replacing half the medium at weekly intervals. Maintainance of CFU-S is not achieved but CFU-C are produced intermittently during the culture period. He agreed that CSF production in a culture of thymus cells alone is high. The system appears to have achieved growth of haemopoietic cells for longer than has been claimed by any other workers.

Metcalf, Van den Engh and Price (Toronto) presented information concerning the nature of colony stimulating factors. Molecular weights of between 15000 and 45000 daltons, depending upon the source, were reported by Metcalf. All are neuraminic acid-containing glycoproteins and cannot be degraded to smaller molecules and still retain activity. Nevertheless they share common functional activities. Van den Engh described variations in physico-chemical properties of CSF depending upon the source from which it is obtained. Those made from human urine, pregnant mouse uteri and fibroblast-conditioned medium have been shown to have similar dose response curves and similar maximum colony stimulating activities. Therefore he, also, suggested that the action of these different CSFs is identical. By experiments in which CSFs from different sources were mixed in various proportions he had shown that their activity is simply additive. On the other hand serum from endotoxin-treated mice behaves differently, due to the presence of a colony-enhancing factor which augments the action of CSF but cannot act alone. A similar enhancer is found in rat red cell lysates.

Working with colony-stimulating activity (CSA) prepared from human leucocyte conditioned medium (LCM) Price showed that it is heterogeneous in both physical and biological properties. Two major physical classes can be recognized, a high MW-CSA (MW about 35,000) and a low MW-CSA (MW less than 1330). High MW-CSA prepared from normal or leukaemic leucocytes stimulates uptake of $^3$H-thymidine in cultures of human leukaemic blast cells. Low MW-CSA from normal human leucocytes stimulates both normal progenitors and those from leukaemic patients in remission, whereas the analogous material from leukaemic leucocytes affects only leukaemic cells and cells from patients relapsing after chemotherapy. The specificity of these CSAs from different sources was further confirmed by experiments performed to demonstrate specific binding to cells related to the source. These data extended the work reported by McCulloch postulating the production of stimulating factors of different specificity from normal and leukaemic cell populations.

Further work on leukaemic cell populations was presented by Mak (Toronto). He reported the finding of an RNA-dependent DNA polymerase (reverse transcriptase) in cells from 6 leukaemic patients in relapse and in small amounts in 8 patients in firm haematological remission. The leukaemias examined included AML, ALL and CML. An increase in the amount of reverse transcriptase was found after culture of these cells for 7 days even without conditioned medium. When cultures were stimulated with medium conditioned by cells from a patient with haemachromatosis the levels rose 1·7 to 13 times in the case of patients in relapse and 3 to 90 times in cultures of cells from patients in remission. When the endogenous reverse transcriptase activities were analysed on sucrose density gradients two discrete homogeneous bands appeared containing (a) 70*s* RNA complex and (b) endogeneous DNA polymerase activity yielding DNA-RNA hybrids as products. These properties are consistent with those described for leucoviruses. Further, preliminary EM studies have revealed the presence of C-type particle-like structures in these bands.

The complexity of the stimulating activities found in leucocyte-conditioned medium was revealed more clearly in discussion when it was stated that stimulation of colony formation, thymidine incorporation and reverse transcriptase stimulation are not necessarily all elicited at the same time.

A group of contributions from Frindel (Paris), Schofield (Manchester) and Tridente (Verona) attempted to demonstrate cooperation between cells from the thymus and some bone marrow cells. Frindel reported findings indicating a response of bone marrow CFU to antigen stimulation in intact mice which does not occur in thymectomized mice. Thymectomy does not lead to any change in CFU regulation leading to erythropoiesis or granulopoiesis since the compartments of erythroid and granulocytic progenitors remain normal. Since antigen response in the adult thymectomized mouse still occurs it appears that CFU stimulation is prevented in these animals because the thymus cannot effect differentiation from the CFU (or early derivatives) in response to feed-back from the antigen-sensitive cells.

Schofield reported experiments demonstrating (as other workers have also) that thymus cells do not inhance spleen colony formation when added to a syngeneic marrow suspension injected into irradiated mice. On the other hand, when live syngeneic thymus cells (but not irradiated ones) are injected into sub-lethally-irradiated mice the number of colonies is increased by about 3 times. When lethally irradiated mice were injected with a mixture of irradiated bone marrow or spleen cells and thymus cells the number of colonies formed is doubled. The increased colony production is not associated with increased CFU numbers in those spleens and the thymus-derived cells do not appear to effect CFV replication. Colony enhancement can be achieved in foetal liver cell suspensions (particularly from very early foetal livers) without the need to irradiate the haemopoietic cell suspensions. It was suggested that a short range cell–cell interaction occurs which leads to greater proliferation of committed cells in the haemopoietic tissues. Only by killing a proportion of the thymus-derived cells normally present in haemopoietic tissues can this cell requirement be revealed.

Tridente showed a great enhancement of $^{3}H$-thymidine incorporation 24 hours after HPA stimulation of a mixture of thymus cells (from 7-day old mice) and adult bone marrow cells, compared with the small effect of stimulation of either population alone. The responding cell is in the thymus population as revealed by experiments in which either the thymus or marrow cells were blocked before stimulation. Attempts have been made to identify the co-operating cell in the marrow by cell separation techniques which reveal that it exists in a very small fraction in the density region 1·065–1·070. It is not associated with the CFU-C nor with the major peak of theta-positive cells. The nature of the collaboration is still not understood but this group of papers, and that by Dexter earlier, pose many questions on the interaction between cells of the haemopoietic and lymphoid tissues.

Extending his studies on differential stimulation of different parts of the haemopoietic system, Reissman (Kansas) reported effects of 19-nortestosterone deconate (19-ND) and erythropoietin in mice treated with bis-chlorethyl-nitrosourea (BCNU). BCNU at a dose of $\sim LD_{10}$ reduces splenic and femoral CFU to 50% of controls. Granulopoiesis recovered within 5 days but recovery of both CFU and granulopoiesis was markedly accelerated by daily injections of 19-ND with no detectable effect on erythropoiesis. This treatment resulted in granulocyte counts up to 10 times normal probably by effects upon the committed granulocytic precursors rather than on CFU recovery. By daily injections of erythropoietin normal erythropoiesis was restored within 3 days whereas without erythropoietin it remained at 20% of control for at least 10 days. The stimulatory effects are presumably on the ERC or pre-ERC populations since no acceleration of CFU recovery was produced by erythropoietin.

Genetic defects (in the grey collie dog and the $Sl/Sl^{d}$ mouse) were the basis of studies reported by Patt (San Francisco) and Wolf (Seattle), respectively. The cyclic reduction in neutrophil, reticulocyte and platelet production occurs every 12 days but there is a phase difference between granulocyte depletion on one hand and reticulocyte and platelet reduction on the other. This, Patt suggested, is due to a restricted stem cell population, either in numbers, or functionally because of high stimulation thresholds. The periodicity of the cell production was postulated to be determined primarily by the neutrophil demand with secondary effects on the other cell types. By increasing neutrophil demand with prolonged endotoxin treatment, neutrophil oscillation (and eventually reticulocyte and platelet fluctuations to a large extent) were eliminated but returned when treatment was stopped. While the actual defects and mechanisms are not clearly understood this dog provides a unique situation for the investigation of haemopoietic control mechanisms.

Wolf, furthering his observations on the stromal environment of the haemopoietic tissues showed that the $Sl/Sl^d$ mouse permitted only restricted proliferation even when associated with normal tissue. Grafting normal spleen onto $Sl/Sl^d$ spleen or vice versa, *in vivo*, left each half spleen exhibiting its own characteristic support of haemopoiesis irrespective of whether the host was a normal or $Sl/Sl^d$ mouse. Lodgement of injected CFU into an irradiated $Sl/Sl^d$ appears normal since on transfer of the CFU from the spleen into an irradiated normal mouse a normal cross-section of colony types is obtained. This experiment was criticized on the basis of the time of transfer which preceded the final settlement of the CFU. Implants of normal splenic or marrow stroma (even when irradiated with 1300 rad) into $Sl/Sl^d$ spleen stimulate erythropoiesis in the immediate vicinity of the implant. Whilst a dispute still continues between microenvironmental versus stochastic determination of differentiation some of the data presented by Wolf were compelling.

The disruption of haemopoietic control brought about by infection of a mouse with the Rauscher virus was discussed by Seidel (Ulm). The effect upon the CFU-S population is to increase turnover rate and this is accompanied by elevation of the CFU-C population by as much as 30 times after an initial depression. Erythroblastosis is also a characteristic feature of the Rauscher virus infection leading to the suggestion that the pluripotent stem cell is probably the target of the virus, with resulting increased differentiation and turnover. However, the maturation capabilities of the granulopoietic cells and of CSF production are both impaired during the course of infection and leukaemia.

Byron (Baltimore) described work, performed in Manchester, on the induction of CFU-S into cycle by various steroids, dibutyryl cyclic-AMP and isoprenaline. He described experiments which show that the site of action is on $\beta_1$ or $\beta_2$ adrenergic receptor sites, depending upon the compound used; and that the mechanism of initiation is through the adenyl cyclase system in the cell. Other experiments show that acetylcholine and carbamylcholine can also initiate DNA synthesis in $G_0$ CFU-S and these compounds appear to operate through cholinergic cell surface receptors. It is of interest that the compounds, whilst tested in short term *in vitro* cultures, operate at physiological concentrations. So far nothing is understood of how these actions can regulate cell population size but the work described provides the first hint of the mechanism of haemopoietic regulation at the level of the individual stem cell.

The second part of the meeting was devoted entirely to the problem of specific proliferation inhibitors ("chalones"?) in the control of haemopoietic tissue. It fairly quickly became obvious that different mechanisms were involved in the various systems described and consequently, rather than use the word " chalone " and all the connotations that the word invokes, the term " Specific Inhibitor " will be used throughout this report.

Houck (Washington) dealt with the effectiveness of lymphoid tissue extracts in inhibiting the incorporation of tritiated thymidine ($^3$H-TdR) by proliferating cell systems. He demonstrated the same degree of effect, irrespective of the source of the extract (thymus, spleen, lymph node, blood), on both T and B lymphocyte populations. He found this effect to be independent of the method of lymphocyte stimulation. These observations were all made *in vitro* but later, Kiger (Villejuif) showed that although the specificity of the thymus cell extract, *in vitro* is not strikingly different for either T or B lymphocytes, *in vivo* the thymus extract shows a clear specificity for T cells in that T cell immune reactions (*e.g.* PHA-lymphocyte stimulation, graft versus host reaction and T and B cell co-operation immune reactions such as antibody formation) are depressed while antibody formation against B cell triggers are not affected. She therefore concluded that two lymphoid inhibitors exist.

Houck claimed that the molecular weight of these inhibitors is in the region of 30,000 to 50,000 daltons and suggested that Kiger's value of about 20,000 daltons is probably due to an artefactual difference produced by differences in methodology, *i.e.* between Sephadex column chromatography and molecular ultrafiltration.

On the question of binding of lymphoid extracts to lymphoid cells, Houck showed that B cells from a human lymphoblastic tumour cell line are able to produce as much inhibitor as cells from any other lymphoid tissue but are considerably less able to retain the applied extract than are normal cells. They are thus released from inhibition more easily, a finding which corresponds to that of Rytömaa for chloroleukaemic cells.

The problem of possible interaction between PHA and lymphoid extracts was overruled by the fact that other mitogens behave similarly to PHA. More important, extracts are effective on the lymphoblastic cell line which is highly proliferative without any mitogen.

In relation to the use of lymphoid extracts for suppression of GvH reaction, Houck reported that 5 mg of splenic lymphoid extract, 3 times/week increases skin graft survival times from 10·5 days to 29 days, the inhibitor treatment stopping after 21 days because of shortage of material. Similar muscle extracts have no effect on the survival time.

Garcia-Giralt (Villejuif) devoted himself entirely to the question of GvH suppression and demonstrated that treatment of parental spleen cells (*in vivo* or *in vitro*) with a partially purified spleen extract (S4) increases the survival time of lethally irradiated Fl hybrid mice compared with mice injected with untreated parental cells. The mechanism of this effect was thought to be that the spleen extract prevented the parental spleen cells from responding to the antigenic stimulation provided by the hybrid recipient. However, irradiation of S4 treated mice or S4 treatment of irradiated mice induces no differences in the lymphoid organs during repopulation compared with the non S4 treated animals. At the same time, splenic lymphocytes from the S4 treated regenerating mice have a decreased PHA or pokeweed mitogen transformation rate. Thus, he came to the conclusion that S4 does not inhibit the proliferation of lymphocyte precursors but only the target cells, the immune-competent cells.

Questions were asked concerning the toxicity of the extracts. It was suggested that for something which should essentially provide a short term effect, long term GvH suppression is surprising. Garcia-Giralt admitted this but suggested that lymphoid extracts bind to the cells preventing transformation. A similar question arose later in connection with the statement by Laerum that incubation of bone marrow cells with granulocyte extracts before plating in agar for CFU-C assay causes a 30–50% loss of colonies measured 7 days later. It was found difficult to equate this with anything other than a direct cytotoxic effect on the CFU-C although Laerum said the effect can be reversed by washing the cells before plating.

Rytömaa (Helsinki) introduced the granulocyte inhibitor session with a summary of much of his early work illustrating reduced $^{3}$H-TdR labelling indices of granulocyte precursors in short term bone marrow cultures. Cell proliferation was shown to be severely damped while RNA synthesis was not affected. He further demonstrated a reduced cellularity and $^{3}$H-TdR incorporation of normal and chloroleukaemic granulocytes grown in diffusion chambers implanted in rats bearing chloroma tumours, the effect being produced by a continuous infusion of inhibitor released by the tumour.

Using the superior qualities of an *in vivo* culture technique afforded by the diffusion chamber (DC) method, Benestad (Oslo) first demonstrated some of the characteristics of DC cell populations. He found that cell growth is inhibited by large cell densities after 2–4 days, mainly due to the action of local DC factors. These he tried to mimic using granulocyte extracts and found that $^{3}$H-TdR incorporation by proliferating granulocytes is reduced while there is no effect on the proliferating immunoblasts and macrophages. Paukovits (Vienna) later showed by bone marrow cell fractionation studies that inhibition occurs in all stages of the maturing granulocytic cell series.

Benestad further showed that injection of hydroxyurea, a specific S-phase killing agent, to DC bearing animals reduces the cellularity of the DC to approximately 50% of control. A prior injection of granulocyte extract is able, presumably by S phase suppression or by blocking entry to S, to protect the cells from the toxic effects of hydroxyurea and approximately 80% of the cells survive. However, because macrophages are also spared by the inhibitor, he emphasized the need to start extracting from pure cell populations. This reinforced a question raised earlier in connection with the lymphoid extracts as to the evidence that the active extract comes from the lymphocytes in view of the fact that the lymphoid organs contain such mixed cell populations.

Laerum (Oslo) and Maurer (Tübingen) employed a new fluorometric technique which measures the DNA content of single cells, 50,000 cells being measured in the space of 2 minutes and being plotted out in the form of a histogram of DNA content. Using this method they were able to show effects of granulocyte extracts on the numbers of cells in the S and $G_2$ phases of a cell cycle. With treatment *in vivo*, they claimed a direct effect on cells at the $G_1$–S boundary while treatment *in vitro* shows, in addition, an effect on cells in the S phase. Consequently, as with Kiger's results for the thymic inhibitor, they concluded that one cannot directly compare the *in vitro* and *in vivo* measurements and that the latter give the more specific effect.

Paukovits (Vienna) was the only contributor to investigate the chemical nature of the extracts and to attempt any high degree of purification. Starting from the spleen, he made extracts with ethyl alcohol-acetone mixtures and then made sequential purifications by column chromatography on Sephadex G-25, Sephadex G-15 and Biogel P-6, checking the inhibitory activity at each stage on a short term bone marrow culture. These steps were followed by paper electrophoresis and thin layer chromatography. He affirmed that the inhibitor has a molecular weight of around 2000 to 4000 daltons as originally reported by Rytömaa and the resultant material from his purification procedure was strongly inhibitory in terms of $^3$H-TdR uptake but was not quantitated. He was unable to detect any peptide material in these samples even with detection methods working at the $10^{-12}$ mol level. On the other hand, he could inactivate them with proteolytic enzymes such as protease, chymotrypsin or papain which clearly do indicate the peptide nature of the purified extracts

Paukovits further warned that thymidine is a constituent of primary granulocyte extracts and this may be a complicating factor in interpreting thymidine incorporation data. This comment highlighted a recurrent question throughout the day concerning the effects of thymidine which seems to be present even in some partly purified fractions of cell extracts. Houck claimed it is a feature of whether the extract is made from fresh tissue or from acetone extracted tissue powders. Rytömaa made estimations of the thymidine content of his extracts to show that very low levels existed and reported experiments adding extra thymidine to show that, in his case, thymidine contamination is not a problem.

Less information was available on possible erythroid inhibitors although Bateman (Sussex) demonstrated autoradiographic evidence of effects in short term bone marrow cultures which supported the early work of the Finnish group on this subject. The labelling indices and grain count distributions of erythroid cells were reduced by application of the extract while those of myeloid and lymphoid cells were unaffected. On the other hand, leucocyte extracts did not affect erythroid cells.

Peschle (Naples) studied erythroid inhibitors in a clinical situation with cases of pure red cell anaemia (PRCA). Looking at the serum of these patients he found an inhibitor to erythropoiesis which is localized in the IgG serum chromatographic fraction. During remission produced by immunosuppressive therapy, this inhibitor disappears. He concluded that PRCA is an autoimmune disease characterized by serum IgG inhibitors to erythropoiesis exerting a cytotoxic effect at the erythroid marrow level, sometimes also acting by neutralizing the circulating erythropoietin.

Throughout these sessions, most speakers agreed that the question of specificity of action of the tissue extracts is of the utmost importance. In most cases, specificity was ascertained by taking similarly prepared extracts from unrelated tissues for comparison and/or testing the extracts in question in other cell systems. For example, Houck made brain, muscle and kidney extracts and made tests on fibroblast, HeLa and choriocarcinoma cultures in addition to his lymphoid extract/lymphoid culture system. Garcia-Giralt tested the spleen extract on CFU-S; Kiger, the thymus extract on liver slices. Rytömaa put mastocytoma or HeLa cells in DC as comparison with granulocytic or chloroleukaemic cells. Laerum used skin extracts in comparison with granulocyte extracts. None of these comparisons was acceptable to the sceptical members of the audience because of the basically different nature of the tissues involved. In some cultures (Rytömaa and Bateman), a close relationship between the cell types tested (erythroid and granulocytic) was maintained but doubt was expressed as to the validity of measurements in these cultures because of the well recognized " unhappiness " of erythroid cells with any culture system known.

These problems were, at least in part, resolved by the final paper of the session given by Lord (Manchester). Recognizing the need to define specificity of action of these inhibitors in a clear cut manner he described a method based on the phenomenon of fluorochromasia coupled with the technique of fluorescence polarization for measuring the Structuredness of the Cytoplasmic Matrix of the cells (SCM). This technique has been adapted and developed in the Paterson Laboratories by Drs L. and B. Cercek for studying the action of various agents on living cell populations. The SCM of a cell is a measure of the biophysical state of the cytoplasm of a cell and Lord demonstrated how it varies as a proliferating cell proceeds through its generation cycle. Measurements of the average value of SCM for any population of cells is characteristically low if it is an asynchronous, proliferating population but high if its rate of proliferation is low or non-existent. He then chose to test out the specificity of the three closely related haematological cell extracts (erythroid, granulocytic and lymphoid) on correspondingly closely associated cell systems (foetal liver normoblasts, granulocytic cells in the culture system earlier described by Dexter and PHA stimulated lymphocytes). In all three systems, the only extracts to increase the SCM are those taken from cells of the same type as those measured. Furthermore, for each set of extracts, except possibly the erythroid ones, the effective extracts were those in the molecular weight ranges reported by Houck and Rytömaa. He thus demonstrated a completely clear cut definition of specificity of action of haemopoietic cell extracts but warned that this may not be related to normal physiological control mechanisms.

On the question of toxicity of the extracts which had arisen at various times in the meeting Lord was further able to demonstrate that at the dose level used, the effect is reversible simply by washing the treated cells. Finally, he pointed out that the extracts were made from bovine and rat sources while the test cells came from human and mouse sources. Thus, another criterion of the " specific inhibitor concept ", that of non-species specificity, could be demonstrated.

The content of this symposium, when compared with a similar one held in the same laboratories 6 years ago, indicates the rapid advances made in this field in the past few years. The understanding of control of cell populations is fundamental to the understanding of the mechanisms of malignant processes and it is clear that in the haemopoietic tissues major progress has been achieved. This symposium, of course, revealed large areas which are, as yet, not understood and in which techniques are not available for investigation. An encouraging feature, on the other hand, was the beginning of transfer of information and techniques from experimental animals to the study of human disease. The final impression is that the momentum of research in this field by relatively few workers is encouraging and one might anticipate that a much clearer understanding of the mechanisms of control of haemopoietic populations and their involvement in haematological disorders will be available within the next few years.