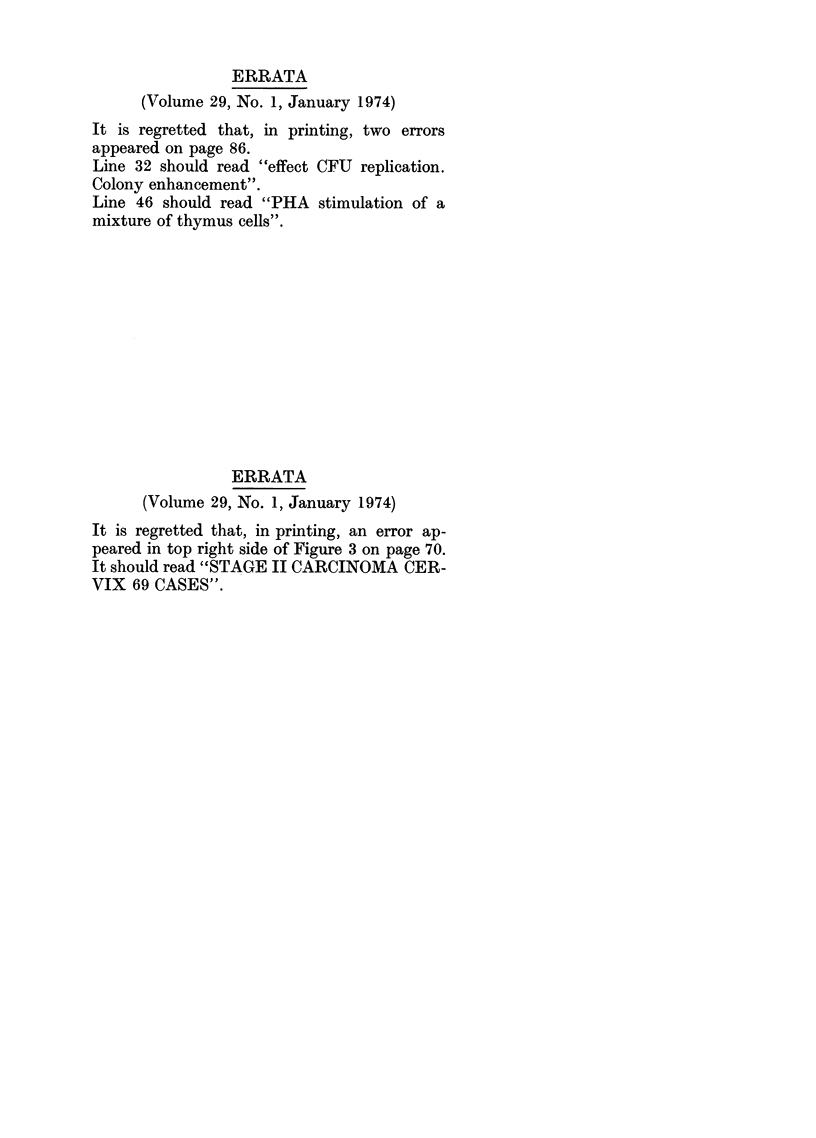# Errata

**Published:** 1974-01

**Authors:** 


### ERRATA

(Volume 29, No. 1, January 1974)

It is regretted that, in printing, two errors appeared on page 86.
Line 32 should read "effect CFU replication. Colony enhancement".
Line 46 should read "PHA stimulation of a mixture of thymus cells".